# Regulation of Mitochondrial Hydrogen Peroxide Availability by Protein S-glutathionylation

**DOI:** 10.3390/cells12010107

**Published:** 2022-12-27

**Authors:** Ryan J. Mailloux, Cathryn Grayson, Olivia Koufos

**Affiliations:** The School of Human Nutrition, Faculty of Agricultural and Environmental Sciences, McGill University, Sainte-Anne-de-Bellevue, QC H9X 3V9, Canada

**Keywords:** mitochondria, bioenergetics, glutathionylation, redox signaling, hydrogen peroxide

## Abstract

Background: It has been four decades since protein S-glutathionylation was proposed to serve as a regulator of cell metabolism. Since then, this redox-sensitive covalent modification has been identified as a cell-wide signaling platform required for embryonic development and regulation of many physiological functions. Scope of the Review: Mitochondria use hydrogen peroxide (H_2_O_2_) as a second messenger, but its availability must be controlled to prevent oxidative distress and promote changes in cell behavior in response to stimuli. Experimental data favor the function of protein S-glutathionylation as a feedback loop for the inhibition of mitochondrial H_2_O_2_ production. Major conclusions: The glutathione pool redox state is linked to the availability of H_2_O_2_, making glutathionylation an ideal mechanism for preventing oxidative distress whilst playing a part in desensitizing mitochondrial redox signals. General Significance: The biological significance of glutathionylation is rooted in redox status communication. The present review critically evaluates the experimental evidence supporting its role in negating mitochondrial H_2_O_2_ production for cell signaling and prevention of electrophilic stress.

## 1. Introduction

Reactive oxygen species (ROS) are by-products of O_2_ metabolism and can induce cytotoxicity through the induction of “oxidative stress” [1,2]. Advances in redox biology methods have identified that oxidants such as H_2_O_2_ can also be second messengers [3,4]. These advances led to remarkable discoveries in redox biology such as identifying mitochondrial H_2_O_2_ as a signal for hypoxia inducible factor-1 (HIF-1) pathway activation [5]. New terms were then developed to extend off the original definition for “oxidative stress” to account for both the signaling and cytotoxic properties of H_2_O_2_. “Oxidative eustress” is a meaningful oxidative stimulus that elicits changes in cell behavior in response to physiological or environmental stimuli [6]. “Oxidative distress” refers to non-specific protein oxidation, tissue damage, and cell death. The difference between the two is related to the concentration of H_2_O_2_: low-to-mid nanomolar concentrations induce eustress signals while distress occurs in the high nanomolar-to-micromolar range [6].

Like other second messengers, H_2_O_2_ signals are modulated by its availability. An acceleration in H_2_O_2_ production occurs through nutrient metabolism and electron transfer reactions. Its availability can be subsequently decreased by antioxidant defenses and/or the inhibition of its production. The availability of H_2_O_2_ ultimately depends on NADH and NADPH availability, central electron carriers in the cell “Redox Code” [7]. Mitochondria are an important source of cell H_2_O_2_ and use this non-radical as a “mitokine” for cell communication [8]. Mitochondria can contain up to 16 H_2_O_2_ sources, with some, such as complexes I and III, serving as integral platforms for mediating redox signals [6,9,10]. Pyruvate dehydrogenase (PDH) and α-ketoglutarate dehydrogenase (KGDH), entry points for monosaccharides and amino acids into the Krebs cycle, also exhibit high rates for H_2_O_2_ production and have been implicated in redox sensing and signaling [11,12]. Other sources, such as dihydroorotate dehydrogenase (DHODH), may also fulfill this role [13].

Hydrogen peroxide elimination is facilitated by catalase and NADPH-dependent antioxidants such as the thioredoxin (TRX) and glutathione (GSH) systems. For glutathione, peroxidases oxidize two molecules of GSH to remove H_2_O_2_, producing glutathione disulfide (GSSG) [14]. GSH is then reactivated through the reduction of the disulfide bridge in GSSG by reductases in the presence of NADPH [15]. GSH occurs in the millimolar range in mammalian cells with a GSH/GSSG of ~100 throughout the cell with the exception of the lumen of the endoplasmic reticulum [16]. In addition, ~10% of the cell’s GSH content is in mitochondria. GSH/GSSG is subjected to H_2_O_2_ and NADPH-dependent spatiotemporal fluctuations at the subcellular level, which can impact various signals such as proliferation, growth, migration, and differentiation [6].

In the early 1980s, it was found that fluctuations in GSH/GSSG can result in the covalent modification of cysteine residues called “protein S-glutathionylation” [17]. Recent experimental evidence using 16 different cell lines demonstrated that ~0.5–1% of the GSH pool was associated with proteins when the GSH/GSSG is ~100 and decreasing this ratio with oxidants induced a ~50% increase in the total number of protein glutathionyl adducts [18]. Approximately 2200 glutathionylation targets have been identified in mammalian cells (called the “glutathionylome”) and protein glutathionyl adduct formation is required for toxin elimination, locomotion, embryonic development, neural activity, immune cell activation, and many other functions [19,20,21,22,23,24]. This is achieved through reversible glutathionylation of enzymes and proteins required for many cell functions such as cytoskeletal dynamics, gene expression, energy metabolism, ion homeostasis, and signal transduction to name a few [23,24,25,26,27,28].

The available experimental evidence has also shown that glutathione pool oxidation results in the inhibition of H_2_O_2_ production [29,30,31,32,33]. These studies indicate that it serves as a negative feedback loop to prevent H_2_O_2_ mediated oxidative distress whilst regulating its availability for signaling. The aim of this review is to highlight contributions of glutathionylation towards (1) preventing H_2_O_2_ stress, and (2) deactivating H_2_O_2_ signaling. This discussion will be coupled to a historical view of mitochondrial glutathionylation reactions and recent evidence demonstrating that disruption of these circuits may contribute to the development of a myriad of disorders related to dysfunctional protein S-glutathionylation.

## 2. Protein S-Glutathionylation

Sulfur has a large and polarizable electron cloud making cysteine thiols ideal for redox regulation of proteins. Glutathionylation can proceed spontaneously through a simple disulfide exchange reaction between a proteinaceous sulfur and GSSG, resulting in the formation of a protein glutathione mixed disulfide (PSSG) [34]. These reactions are non-specific and require ionization of a cysteine thiol (-SH) to a nucleophilic thiolate anion. However, most protein thiols have a pKa of ~8.5 and are thus non-reactive in neutral environments [23,35,36]. Spontaneous glutathionylation of most cell protein thiols proceeds nonetheless but only during oxidative distress and with increasing probability as the GSH/GSSG approaches ~1 (e.g., when GSSG is abundant). The spontaneous and non-specific modification of proteins with glutathione is a biomarker for oxidative distress and occurs in many pathologies [37,38,39,40,41,42,43]. Reversing glutathionylation reactions and restoring the cell glutathionylome by preserving a high GSH/GSSG is a potential avenue for treating several disorders such as fatty liver, cataract formation, atherosclerosis, obesity, diabetic cardiomyopathy, type 2 diabetes, and age-related sarcopenia [22,42,43,44,45,46,47,48,49].

Most protein cysteine residues are resistant to spontaneous glutathionylation unless the concentration of GSSG is high. However, not all proteinaceous thiols share this characteristic. Exposure of solvent accessible cysteines to a slightly alkaline subcellular milieu increases the probability for thiol ionization. For example, the basic pH of the mitochondrial matrix lowers thiol pKa, promoting glutathionylation [50]. Surrounding cysteines in a microenvironment consisting of basic amino acids can also increase the likelihood of thiolate formation, such as in the glutaredoxin-1 (GRX1) active site, which contains a catalytic thiol with a pKa of ~3.5 [34]. Most cell protein thiols have also been reported to only be modified when the GSH/GSSG is ~1 [51]. However, certain proteins such as c-Jun can be modified when the glutathione pool is only slightly oxidized, meaning they are more likely to undergo spontaneous glutathionylation [51]. There is also the new phenomenon of *cryptic glutathionylation* sites where exposure of a buried cysteine reveals a thiol amenable to ionization and spontaneous modification with glutathione [52,53]. This concept was first reported in 2014, when it was found that stretching of titin protein in muscles and the resulting unfolding of its Ig-domains reveals modifiable cysteines that can be glutathionylated by GSSG [52]. A spontaneous modification by glutathionylation may also occur following the oxidation of thiolate anion (Pr-S^−^) by H_2_O_2_. This mechanism for glutathionylation has been reviewed extensively elsewhere, but is also reliant on thiol ionization since the peroxide-mediated oxidation of cysteine sulfur has activation energy restraints (spontaneous oxidation of a thiol by H_2_O_2_ is ~10^1^ M^−1^ s^−1^, which is several orders of magnitude lower than its rate of elimination by peroxidases (see below)) [54].

Evidence collected in the 1980s demonstrated that glutathionylation is integral for protecting cysteines from oxidative deactivation, which can be reversed by the catalytic activity of a thiol oxidoreductase, later identified as GRX1 [17,55,56]. The regulatory function of glutathionylation was later defined by Wang et al. in a study showing G-actin polymerization was inhibited by glutathionylation [57]. This was reinforced later by Pastore et al. where it was found that aberrant increases in glutathionylation of actin correlated with Friedrich’s ataxia [27]. Growth factor signaling induced H_2_O_2_ generation by NADPH oxidase, leading to glutathione pool oxidation and modification of monomeric actin [57]. Reduction of the pool had the opposite effect: promoting deglutathionylation in a reaction driven by GRX1 [57]. These seminal findings led to the discovery that reversible glutathionylation of monomeric actin in response to H_2_O_2_ signals was vital for the induction of neutrophil chemotaxis, adhesion, phagocytosis, and neutrophil extracellular trap (NET) formation, processes integral to immune cell function [58,59].

There are many redox modifications that occur on cysteines, such as nitrosylation, disulfide formation (intra- or intermolecular bridge), sulfenylation, and persulfation [6]. We direct the reader to excellent reviews on these modifiers and their importance in cell physiology [6,22,60,61]. Here, we will only be discussing glutathionylation reactions. As a regulatory element, protein S-glutathionylation was the first redox modification found to meet the requirements of a post-translational modification (PTM) for protein regulation in response to stimuli [25]. Other redox modifications, such as S-nitrosylation, which involves the addition of a nitric oxide moiety to a protein thiol, may also fulfill these requirements. Indeed, an S-nitrosylation motif was identified in proteins and inducible nitric oxide synthase (iNOS), when bound to the calcium-chelating proteins S100A8 and S100A9, can drive nitro-modification reactions (reviewed in [62]). Additionally, sulfenylation reactions can also alter protein functions if the resulting P-SOH group can be stabilized by the surrounding microenvironment [63]. However, most H_2_O_2_ signals occur through disulfide bridge relay reactions catalyzed by peroxiredoxin-thioredoxin systems or S-glutathionylation [10]. Persulfation driven by H_2_S can also play a regulatory role and it was recently shown that a mitochondria-targeted sulfide generator can protect from ischemia-reperfusion injury through modification of cysteines in the electron transport chain [64]. Many other H_2_S targets have been identified and so it is a potential regulator of proteins through cysteine oxidation.

**Figure 1:**
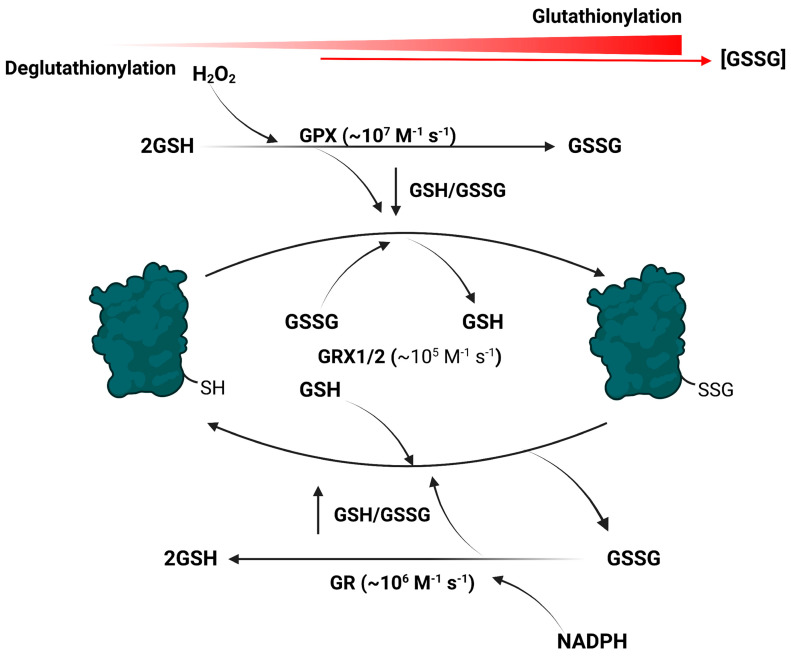
The addition and removal of glutathione to and from target proteins is a rapid event that occurs in response to spatiotemporal fluctuations in GSH and GSSG availability. Cell nutrient metabolism and electron release from various enzymes (e.g., flavin-dependent dehydrogenases) causes the rapid production of H_2_O_2_ and its clearance by glutathione peroxidases (GPX). This results in the oxidation of the glutathione pool and a decrease in the GSH/GSSG ratio. The forward glutathionylation reaction is driven by glutaredoxins (GRX1; cytoplasm and intermembrane space, GRX2; matrix) in response to glutathione pool oxidation. Production of NADPH by the same nutrient oxidizing pathways that generate H_2_O_2_ is used by glutathione reductases (GR) to reduce the disulfide bridge in GSSG, reforming GSH and restoring the reducing power of the glutathione pool. This activates the deglutathionylase activities of glutaredoxins, resulting in the removal of GSH from a target protein. Figure was generated with Biorender.com, accessed on 1 June 2022.

Reversible glutathionylation is highly specific and the reactions occur in response to physiological/environmental stimuli [65]. Recent advances in redoxomics for assessment of the cell glutathionylome have also identified the presence of so-called conformation-dependent cryptic cysteines, like in titin (discussed above), and glutathionylation can also occur on amino acid residues that have undergone the β-elimination of H_2_O or phosphate (called protein C-glutathionylation where a thioether linkage is formed between GSH and a dehydro-amino acid, reviewed in [53]). In mammalian cells, reversible glutathionylation occurs in response to changes in glutathione pool redox buffering capacity (Figure 1). Glutaredoxins (GRX1: cytoplasm and intermembrane space, and GRX2: mitochondrial matrix) drive glutathionylation in response to GSH oxidation whereas the deglutathionylase activity is activated by reduction of the pool (Figure 1). The second-order rate constant for the reaction of GRX with GSSG is ~10^5^ M^−1^ s^−1^, with kinetics for GRX-mediated glutathionylation of its active site and subsequent transfer to proteinaceous thiols increasing with increasing GSSG availability [66,67]. The deglutathionylation of protein targets occurs at up to 10^5^ M^−1^ s^−1^ as well [50,68]. Further, one must factor in the rapid kinetics for glutathione peroxidases (~10^7^ M^−1^ s^−1^) and glutathione reductases (~10^6^ M^−1^ s^−1^) (Figure 1) [69,70]. Together, the reversible glutathionylation of proteins in mammalian cells in response to GSH pool oxidation and reduction can elicit rapid changes in cell behavior. Glutathione S-transferase (GST) isoforms GSTP and GSTM also drive glutathionylation of several cell targets at a high rate and has been implicated in modulating energy sensing, purine metabolism, and endoplasmic reticulum function [66]. GSTO-1, by contrast, can deglutathionylate target proteins at high rates [66]. Recent work has established GST can glutathionylate xanthine oxidoreductase in rat liver cytoplasm, resulting in decreased H_2_O_2_ production by the enzyme [32]. This mechanism would suggest that GST, like the GRXs, is required to “manage” the cell H_2_O_2_ budget by inhibiting its production. Less is known about GST-mediated reversible glutathionylation. However, it is clear it is vital for the redox regulation of proteins and may also be integral for desensitizing cell H_2_O_2_ signals.

## 3. Mitochondrial Glutathionylation Reactions

Mitochondria contain a high percentage of a mammalian cell’s glutathionylation targets (~30% of the cell’s modifiable proteins). Insights into protein S-glutathionylation and deglutathionylation in response to the fluctuation in GSH availability was first generated through the identification of GRX2 in the matrix of mitochondria and its impact on complex I function [33,71,72]. Oxidation of glutathione pools induces the GRX2-mediated modification of the NDUFS1 subunit in complex I, inhibiting its activity and H_2_O_2_ generating capacity [33,73,74]. Maintaining the glutathione pool in a reduced state has the opposite effect [33,73,74]. Modification of NDUFS1 by glutathionylation has been reported in cardiac, muscle, eye, and liver tissue and it is required to protect complex I from oxidants [73,74,75,76]. Other complex I subunits, such as NDUFV1 and NDUFA11, are also targets [77,78,79]. Modification of NDUFV1 has also been linked to the inhibition of complex I. Recent work conducted on rats found many other components of complex I are subjected to modification following exercise [20]. Additionally, induction of deglutathionylation improves mitochondrial metabolism in cardiomyocytes by maintaining complex I in an active state [80]. Overall, complex I is an important site for the glutathionylation-mediated regulation of mitochondrial bioenergetics in response to redox state altering cell stimuli.

**Figure 2:**
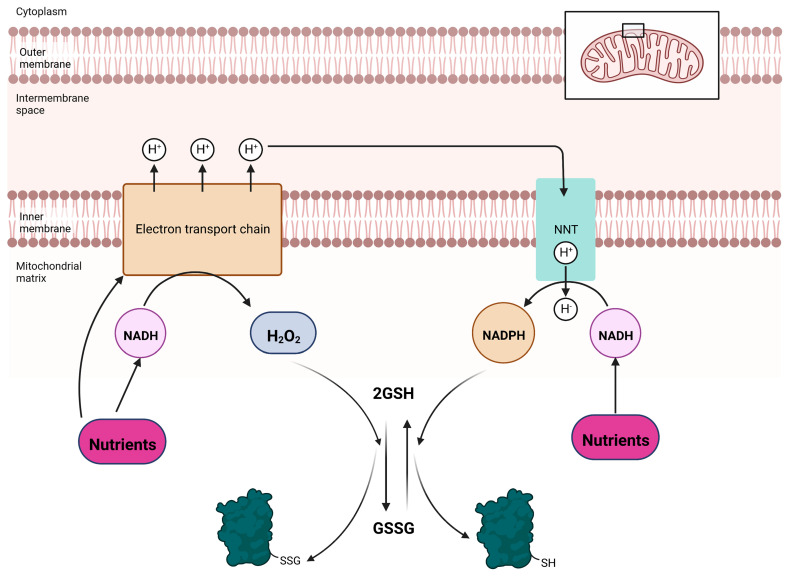
Reversible glutathionylation reactions in mitochondria are intimately linked to oxido-reduction reactions that drive nutrient oxidation and NADPH production. Oxidation of nutrients to produce NADH, either by the Krebs cycle or other non-Krebs cycle reactions (e.g., amino acid catabolism or fatty acid oxidation), fuels electron transfer and the creation of a transmembrane potential of protons. Nutrients can also be oxidized directly by the electron transport chain. Electrons are used to reduce O_2_ at the end of the chain to H_2_O and the protonmotive force (PMF) induces the phosphorylation of ADP by complex V. Electrons originating from these nutrients can also react with O_2_ in several flavin-dependent dehydrogenases and the electron transferring chain to generate H_2_O_2_. Note that sites that generate ROS in mitochondria produce a mixture of O_2_^•−^ and H_2_O_2_, but the latter form dominates over the former. Any residual O_2_^−-^ formed is converted to H_2_O_2_ by SOD. Clearance of H_2_O_2_ oxidizes the glutathione pool driving protein S-glutathionylation. The same electron transferring pathways that generate H_2_O_2_ also generate NADPH, a reducing equivalent required for antioxidant defenses. This is achieved by the mitochondrial redox buffer sentinel, nicotinamide nucleotide transhydrogenase (NNT). The proton gradient yielded from nutrient metabolism is tapped by NNT to drive the transfer of a hydride from NADH to NADP^+^. The NADPH generated is used to reduce the glutathione pool, restoring antioxidant defenses and inducing deglutathionylation.

Several factors make mitochondria an ideal site for redox regulation by glutathione. The matrix environment is alkaline (pH = 8.3–8.5), favoring thiol ionization. The matrix environment is also rich in proteinaceous thiols (60–90 mM) and a large fraction of these are solvent-exposed sulfurs [81]. The glutaredoxins also occur in the 0.5–1 µM concentration [50]. These concepts have been discussed in several reviews, but what has not is how reversible glutathionylation is inexorably linked to bioenergetics (Figure 2). Mitochondrial bioenergetics relies on nutrient and electron transfer reactions, which produce H_2_O_2_. Although superoxide (O_2_^−^) is often viewed as the proximal ROS formed by mitochondria, this may be erroneous given that (1) most mitochondrial sources generate a mix of O_2_^−-^ and H_2_O_2_, with the latter accounting for ~70–80% of the ROS formed [82,83,84] and (2) any O_2_^−-^ formed is dismutated to H_2_O_2_ at 10^9^ M^−1^s^−1^ by superoxide dismutase (SOD1; intermembrane space, SOD2; matrix) [85]. Thus, the proximal ROS formed by mitochondrial bioenergetics is H_2_O_2_. The same nutrient oxidizing and electron conducting pathways that produce H_2_O_2_ also generate NADPH for the reduction of GSSG. Pathways for NADPH production in the matrix include malic enzyme and the isocitrate dehydrogenase isozyme, IDH2. However, the main source for NADPH in mitochondria is nicotinamide nucleotide transhydrogenase (NNT), which uses the protonmotive force (Δp) to drive its production [86]. It is worthy to note that H_2_O_2_ production is linked to the Δp too, where a small increase in protonic backpressure on the respiratory chain results in the exponential acceleration of H_2_O_2_ production [87]. This also means the “uptick” in the strength in the Δp would also power NNT, off setting the H_2_O_2_ mediated oxidation of mitochondrial GSH pools (Figure 2). Taken together, reversible glutathionylation is linked to mitochondrial bioenergetics where the redox buffering capacity of the GSH pool varies in response to H_2_O_2_ and NADPH availability (Figure 2). This makes mitochondrial bioenergetics a powerful determinant for the forward and reverse modification of proteins with a glutathionyl moiety.

For the most part, adding a glutathionyl moiety to a protein inhibits its activity in mitochondria (reviewed in [88,89]). Exceptions including mitochondrial fusion proteins, protein import machinery, and complex II (reviewed in [88]). Glutathione pool oxidation results in the inhibition of respiration and oxidative phosphorylation (OXPHOS), proton leaks-dependent respiration, solute import, and nutrient oxidation and Krebs cycle flux (reviewed in [88,89]). Fatty acid oxidation enzymes are also reported to be deactivated by S-nitrosylation and recent work has demonstrated that promoting mitochondrial deglutathionylation can augment fat oxidation by ~4-fold in muscle tissue [44,90]. Complexes III, IV, and V are also have reported glutathionylation sites and glutathionylation inhibits ATP synthase, which has been related to oxidative-distress mediated heart disease [91,92,93]. However, it remains to be determined if glutathionylation affects complex III and IV activities. Solute anion carrier superfamily targets include uncoupling proteins (UCP)-2 and 3, adenine nucleotide translocase (ANT), carnitine acyl-carnitine carrier (CAC), and pyruvate carrier. Glutathionylation also inhibits apoptosis and the assembly of the mitochondrial permeability transition pore (reviewed in [88]).

## 4. Glutathionylation and Complex I-Mediated H_2_O_2_ Production

**Figure 3:**
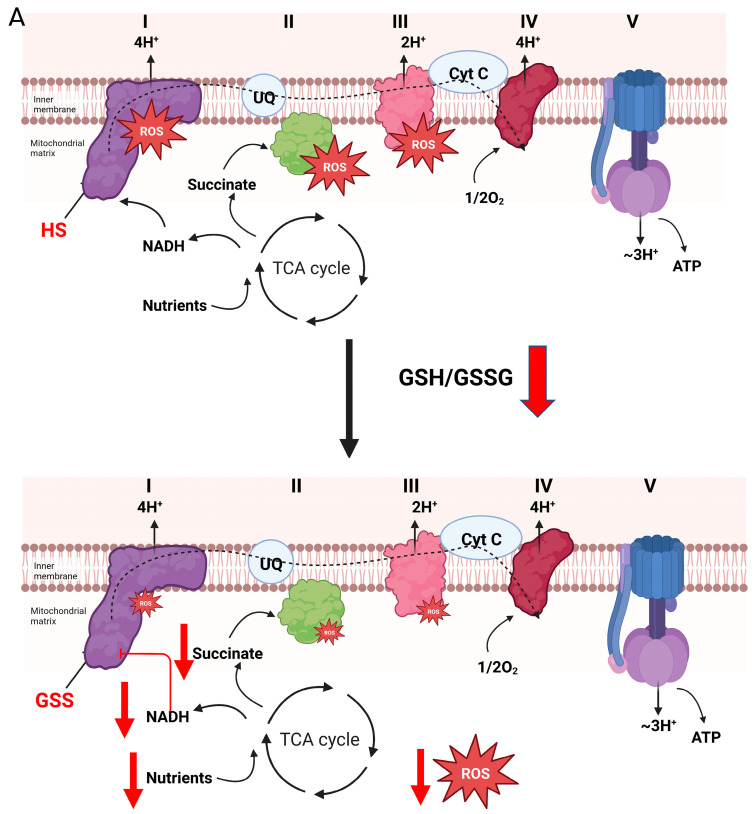

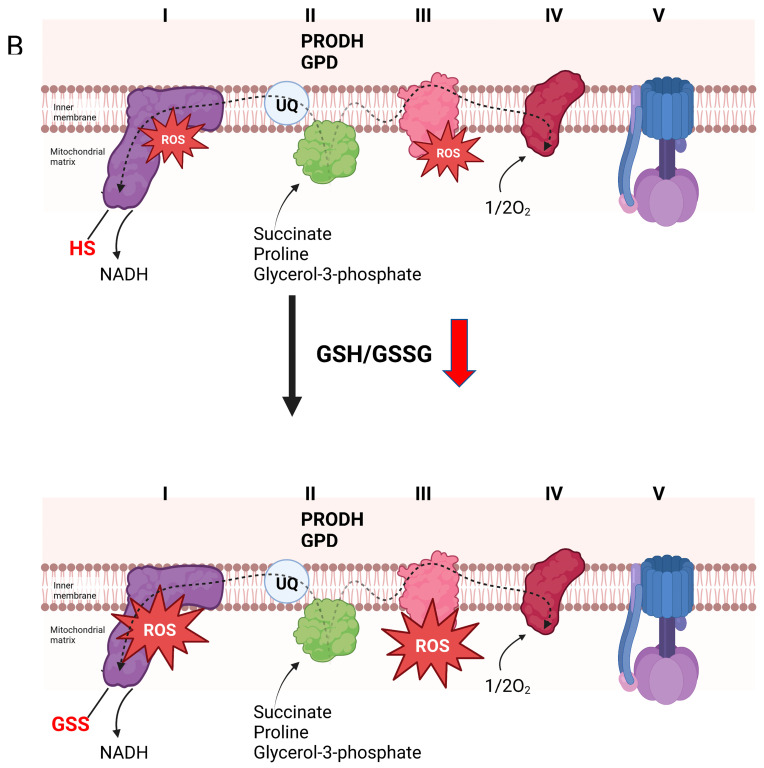
Reversible glutathionylation reactions are integral for controlling the production of H_2_O_2_ by complex I and the electron transport chain. (**A**). Forward electron flow from the Krebs cycle to complex I through the production of NADH (generated by Krebs cycle flux or oxidation of nutrients by non-Krebs cycle enzymes) drives the genesis of both ATP and H_2_O_2_, which is dependent on electron transfer reactions through the respiratory chain. H_2_O_2_ is used in mitochondria-to-cell signaling through direct oxidation of protein thiols or oxidation of redox networks (e.g., glutathione and the reversible glutathionylation of cell proteins). Oxidation of the same redox networks feeds back on sites for H_2_O_2_ production in the respiratory chain resulting in glutathionylation. This inhibits H_2_O_2_ production, decreasing oxidant genesis by mitochondria and promoting the reactivation of mitochondrial redox networks to maintain antioxidant defenses. This mechanism also desensitizes H_2_O_2_ signals emanating from mitochondria during the metabolism of nutrients that generate NADH, which is oxidized by complex I. Blockage of complex I through the glutathionylation of the NDUFS1 subunit inhibits NADH oxidation by preventing electron flow to flavin mononucleotide (FMN), the main site for H_2_O_2_ production in complex I. This mechanism for inhibition of oxidant production also prevents H_2_O_2_ by sites downstream from complex I, such as complex III. (**B**). Over oxidation or prolonged oxidation of glutathione pools results in the extended glutathionylation of target proteins in mitochondria. This can result in increased H_2_O_2_ production of the respiratory chain. Increased H_2_O_2_ production occurs after prolonged glutathionylation of NDUFS1 subunit in complex I. Although this inhibits NADH-mediated H_2_O_2_ genesis, this also promotes ROS production through the oxidation of nutrients that by-pass the Krebs cycle and donate electrons directly to the UQ pool. Bioenergetics that favor reverse electron transfer (e.g., over reduction of electron donating/accepting centers in the respiratory chain and a polarized mitochondrial inner membrane) augments H_2_O_2_ by complex I. Aberrant or prolonged glutathionylation of complex I under these conditions also activates H_2_O_2_ genesis during forward electron transfer to complex III. This prolonged glutathionylation of complex I and the subsequent increase in H_2_O_2_ genesis is likely associated with the oxidative distress observed in many pathologies such as heart disease, development of cataracts, and age-related sarcopenia.

Glutathionylation prevents oxidant distress by protecting vulnerable thiols from irreversible oxidation and inhibiting the production of H_2_O_2_. However, it is likely that reversible glutathionylation serves a second function: desensitizing H_2_O_2_ signals through inhibition of mitochondrial oxidant production. Insight into this second potential function can be extracted from studies conducted on the glutathionylation of complex I over the past twenty years. As noted above, H_2_O_2_ production by complex I can be inhibited by glutathionylation during the oxidation of NADH. This does have strong implications for complex I-mediated cell signaling pathways. Hydrogen peroxide genesis by complex I has mostly been associated with the induction of oxidative distress. However, in 2015, Bleier and colleagues demonstrated, using redox fluorescence difference gel electrophoresis, that H_2_O_2_ emitted by complex I induced the selective oxidation of proteinaceous cellular thiols, which led the authors to suggest the respiratory complex fulfilled a redox signaling function [94]. Later work demonstrated that controlled bursts in H_2_O_2_ genesis by complex I was integral for the induction of brown fat thermogenesis and adaptive signaling and induction of longevity in *D. melanogaster* [95,96,97]. A physiological function for adaptive redox signaling was also identified in rodent exercise models, where it was found muscle contraction and relaxation alters the mitochondrial and cellular glutathionylome through bursts in cell H_2_O_2_ production (~2200 muscle proteins were modified with a glutathionyl moiety) [20,98]. Targets included proteins involved in muscle contraction and relaxation, but also complex I and many mitochondrial dehydrogenases required for nutrient metabolism [20]. A follow-up study by the same group found reducing the glutathione pool in aged rats prevents sarcopenia by restoring complex I activity through promotion of deglutathionylation [41]. Induction of deglutathionylation also augments complex I driven respiration and H_2_O_2_ production in muscle tissue [44]. This protected from diet-induced obesity and fatty liver disease by increasing mitochondrial fuel combustion in muscles and the H_2_O_2_-driven induction of adaptive signaling pathways that increase resistance against electrophilic stress [44]. Therefore, the available evidence so far suggests that adding a glutathionyl moiety to complex I for a short period may serve as a “safety valve” to turn down H_2_O_2_ signals, whereas reactivation of H_2_O_2_ emission by the complex is required for adaptive signaling (Figure 3A). Similar findings have been made with complex I in heart and liver tissue: glutathionylation shuts off its activity and H_2_O_2_ generating capacity, which can be reactivated with reducing agents [37,76]. The goal of reversible glutathionylation in this case is to modulate the availability of H_2_O_2_ for second messaging while protecting mammalian cells from oxidative distress (Figure 3A). Thus, when taken in aggregate, the rapid and reversible glutathionylation of complex I protects from H_2_O_2_ stress by limiting its production and mitigating thiol over-oxidation, but also desensitizes mitochondria-to-cell redox signals.

The importance of reversible glutathionylation in regulating H_2_O_2_ availability by complex I is highlighted further by the consequences associated with disrupting this pathway. Rapid and reversible glutathionylation of complex I is vital for controlling H_2_O_2_ availability and preventing the over-production of electrophiles when Krebs cycle-linked nutrients are being metabolized. However, Taylor et al. showed modification of complex I on NDUFS1 and NDUFV1 with glutathione in bovine mitochondrial membrane extracts augments H_2_O_2_ production [99]. This (seemingly) paradoxical effect was postulated later to be due to reverse electron transfer (RET) from the UQ pool to complex I during the oxidation of nutrients that by-pass the Krebs cycle [100]. This posit was based on the position of NDUFS1 and NDUFV1 in complex I and their role in oxidation-reduction of nicotinamides. Both subunits are part of the N-module and thus form the nicotinamide binding site to facilitate electron transfer from NADH to the flavin mononucleotide (FMN) prosthetic group in the complex [101]. Glutathionylation of NDUFS1 disrupts NADH binding, thereby preventing its oxidation and the production of H_2_O_2_ by complex I. However, oxidation of nutrients that by-pass the Krebs cycle and donate electrons directly to the UQ pool may have the opposite effect. Recently published evidence does support this hypothesis. Indeed, two recent studies found NDUFS1 glutathionylation augments H_2_O_2_ production during RET from glycerol-3-phosphate, proline, and succinate, nutrients that donate electrons directly to the UQ pool (Figure 3B) [76,102]. Glutathionylation of NDUFS1 blocks electron flow during reverse transfer from the UQ binding site to the nicotinamide binding site, promoting electron accumulation at FMN and an increase in H_2_O_2_ genesis (Figure 3B) [76,78]. Furthermore, the increased production of H_2_O_2_ during RET from glycerol-3-phosphate, proline, or succinate is not due to the glutathionylation of glycerol-3-phosphate dehydrogenase, proline dehydrogenase, or complex II, which donate electrons from these substrates to the UQ pool (Figure 3B). These findings could account for the observed increase in oxidant production by mitochondria in several pathologies where complex I displays prolonged glutathoinylation due to defects in the addition or removal of glutathionyl moieties to and from proteins. RET from succinate does induce H_2_O_2_ and electrophilic stress causing myocardial dysfunction and disease [103]. Second, heart disease in rodents and humans correlates with complex I glutathionylation and increased mitochondrial oxidant production (Figure 3B) [37,104]. Further, the number of protein-glutathionyl adducts increases in the hearts and muscles of aged rodents, as does the glutathionylation of complex I [41,42]. Reversal of complex I glutathionylation with reducing agents or mitochondria-targeted antioxidants restores its activity, protects from oxidative distress, and restores ATP production [37,41]. Similar observations have been made with cataracts: loss of GRX2 function or high H_2_O_2_ results in complex I glutathionylation and the induction of oxidative distress [43]. Taken together, complex I is an important site for regulation by glutathionylation and it likely plays an integral role in modulating H_2_O_2_ availability for signaling. However, inherited or acquired defects in (de)glutathionylation pathways can also be detrimental since it can prolong complex I deactivation, leading to oxidative distress and cell damage through accelerated H_2_O_2_ production.

## 5. Glutathionylation and H_2_O_2_ Production by Other Flavoproteins

KGDH and PDH have high rates for H_2_O_2_ production in brain, muscle, and liver mitochondria [105,106,107]. Both α-ketoacid dehydrogenases are also deactivated by the oxidation of vicinal thiols in the lipoamide residue of the E2 dihydrolipoamide transcylase subunit [108]. KGDH and PDH serve as entry points for amino acids and pyruvate from glycolysis into the Krebs cycle, respectively. Together with its role in mitochondrial H_2_O_2_ homeostasis, both enzymes were labeled “redox sensors” that control metabolism in response to changes in mitochondrial redox buffering capacity [109,110]. Three separate studies published by the Szweda group showed lipoamide oxidation was accompanied by its glutathionylation [111,112,113]. This group reported that glutathionylation was required to protect these vulnerable vicinal thiols from further oxidation. These seminal observations led to the development of the hypothesis that glutathionylation also served as a feedback inhibitor for H_2_O_2_ production by these α-ketoacid dehydrogenases. Glutathionylation of the E2 lipoamide thiols in response to glutathione pool oxidation decelerates NADH and H_2_O_2_ production by both KGDH and PDH [29,30]. This can be reversed by GRX2, restoring KGDH and PDH activity and H_2_O_2_ genesis. The high H_2_O_2_-generating capacity of both α-ketoacid dehydrogenases has been suggested to fulfill some cell signaling functions. It has been suggested that both enzymes serve as redox sensors that may operate as platforms for H_2_O_2_-mediated cell communication [109]. This signaling may occur through a redox relay with NNT. A study recently showed such a relay may exist between KGDH and NNT in the mitochondria of cardiac tissue, which is required to protect the heart from oxidative distress [114]. Two studies reported PDH may also function in a redox relay with NNT [115,116]. NNT expends the protonmotive force to drive hydride transfer from NADH to NADP^+^, forming NADPH for antioxidant defenses like glutathione [86]. The PDH-NNT redox relay requires H_2_O_2_ production by PDH, which is quenched by NADPH-dependent antioxidants. This dissipates the Δp for NADPH production by NNT, increasing mitochondrial fuel combustion and respiration. This mechanism, however, was challenged because of valid concerns related to the stoichiometry for proton return and NADPH production by NNT [117]. The PDH-NNT relay-mediated increase in respiration would require ~200 H^+^ to be returned per NADPH produced by NNT [117]. As pointed out in [117], it was recently established that the stoichiometry for proton return/hydride transfer is 1:1 [118]. Thus, a role for NNT in fueling such a large increase in respiration is not probable. Important stoichiometric concerns for this modeling aside, it is possible that such a signaling axis may play a role in regulation of protein functions in response to GSH pool oxidation and reduction. As discussed in Figure 2 and above, reversible glutathionylation is dictated by the redox state of the GSH pool, which is a function of H_2_O_2_ and NADPH availability, molecules that dependent on mitochondrial bioenergetics for production. PDH and KGDH are sources for H_2_O_2_, but also generate the NADH required to fuel NADPH production by NNT. The H_2_O_2_ is metabolized by the GSH pool, resulting in GSSG production and the propagation of signals through glutathionylation. Glutathione pool oxidation also drives the feedback inhibition of H_2_O_2_ genesis by KGDH and PDH (and other sources such as complex I, desensitizing the redox signal. By way of extension, NADPH formed by NNT activity restores the reducing capacity of the GSH pool driving protein deglutathionylation. The signaling function of KGDH and PDH could also be extracted from cancer studies. Indeed, targeted inhibition of both enzymes can interfere with cancer progression and cell division [119]. This could be attributed to the disruption of nutrient metabolism pathways required for anabolic reactions. However, it is also feasible it inhibits H_2_O_2_ genesis, which is a vital second messenger for proliferation and adaptive signaling in cancer cells.

Insight into the potential regulatory role of glutathionylation in H_2_O_2_ signaling can also be extracted from a recent publication regarding its effect on DHODH [120]. Mitochondrial DHODH is in the mitochondrial inner membrane and catalyzes the production of orotate for pyrimidine biosynthesis [121]. Oxidation of dihydroorotate reduces the UQ pool for respiration but can also generate H_2_O_2_ [122]. DHODH is over-expressed in cancer cells and is integral for promoting cell proliferation through pyrimidine biosynthesis and H_2_O_2_ signaling [121]. Thus, targeted disruption of DHODH can serve as a means for cancer treatment. It was recently found glutathionylation inhibits DHODH activity and decelerates dihydroorotate-fueled respiration and H_2_O_2_ production [120]. Of interest, as well, is there was a sex dimorphism found in this pathway [120]. Indeed, female liver mitochondria were “more resistant” to the glutathionylation-mediated inhibition of DHODH, which was attributed to a superior redox buffering capacity when compared to males [120]. Indeed, Mallay et al. were the first to discover a sex dimorphic effect in the glutathionylation-mediated regulation of cell H_2_O_2_ production [123] which was reinforced later by a publication from the same group [74]. The targeted inhibition of mitochondrial proteins by glutathionylation for the induction of cancer cell death is not a new concept. It was reported by Pferffele et al. that deactivation of UCP2 with glutathionylation catalysts sensitized drug-resistant promyeolocytic leukemia cells to chemotherapy [124]. Deactivation of DHODH by glutathionylation may serve a similar role by preventing pyrimidine biosynthesis while deactivating H_2_O_2_ induced proliferation signals.

## 6. Conclusions

The cell redoxome is composed of diverse and reversible oxido-reduction reactions that occur on proteinaceous cysteines. It serves as a vital interface for changing cell responses to environmental cues. These cues include toxins, nutritional changes, exercise, acquired or inherited disorders and many other factors. Glutathionylation is a critical part of the redoxome and can affect many cell programs. Dysfunction in reversible glutathionylation due to the disabling of glutathione S-transferases and glutaredoxins, or prolonged oxidation of glutathione pools is associated with several pathologies and disruption of embryonic development.

Here, we have provided a historical perspective on cell and mitochondrial glutathionylation reactions, which we coupled to emerging evidence demonstrating that it is integral for decelerating H_2_O_2_ production. Most of the findings so far have demonstrated that glutathionylation is vital for inhibiting H_2_O_2_ genesis by mitochondria through the deactivation of sources such as complex I, KGDH, and PDH. The effect is to limit H_2_O_2_ formation for the reactivation of NADPH-dependent antioxidant defenses, thereby preventing oxidative distress. Further, the inhibition must be short-lived since glutathione pool reduction is rapid, resulting in the induction of deglutathionylation and reactivation of electron-transferring nutrient oxidation pathways required for energy transduction.

Coupled with this, we presented the most recent evidence showing that disruption of this pathway can have pathological consequences. On one side, prolonged glutathione pool oxidation due to oxidative distress may lead to the unwanted maintenance of mitochondrial energy transducing enzymes in an inactive state. This can lead to the inhibition of oxidative phosphorylation pathways and metabolic inflexibility, but also contribute to oxidative distress through the augmentation of H_2_O_2_ production for extended periods. In this case, we used empirical evidence demonstrating that glutathionylation of complex I subunit NDUFS1 correlates with mitochondrial dysfunction and oxidative distress in several pathologies. Recent findings have shown that this is related to the acceleration of complex I-mediated H_2_O_2_ genesis due to RET following the oxidation and metabolism of UQ linked substrates such as succinate, proline, or glycerol-3-phosphate. A similar effect may occur upstream from the UQ pool where blocking electron flow or mitochondrial proton return to the matrix increases ROS production by complex III.

Hydrogen peroxide signals are mediated through disulfide relay reactions, either occurring through protein disulfide exchange reactions catalyzed by thioredoxins or glutathionylation. Here, we have presented evidence that glutathionylation is also a feedback loop to desensitize mitochondrial H_2_O_2_ signals. This mechanism is highly effective given the rapid kinetics for glutathionylation/deglutathionylation reactions and their dependence on mitochondrial bioenergetics. These characteristics make glutathionylation ideal for quelling complex I signals and redox communication emanating from other H_2_O_2_ forming dehydrogenases. However, use of glutathionylation/deglutathionylation to modulate H_2_O_2_ signals demands that the addition and removal of GSH to and from proteins is efficient so as to avoid prolonged disruptions in mitochondrial energy transduction pathways.

## 7. General Significance

The glutathionylation and deglutathionylation of cell proteins is linked directly to the oxidation and reduction of the glutathione pool. Cell glutathione availability, in turn, is directly influenced by H_2_O_2_ and NADPH availability, products of mitochondrial respiration and nutrient metabolism. The elimination of H_2_O_2_ by glutathione and the reduction of GSSG in an NADPH-specific manner occurs rapidly, which is equally matched by the fast addition and removal of glutathionyl moieties to and from proteins by GRXs and GSTs. Here, we have discussed the role of glutathionylation in the prevention of oxidative distress in a historical context. This included elaborating its function in protecting thiols from irreversible oxidation and limiting H_2_O_2_ availability. We also discussed its importance in serving as a H_2_O_2_ signal amplifier for the regulation of many cell functions in the contexts of physiology and development and how disrupting protein glutathionyl moiety addition and removal reactions results in pathologies and aging. Coupled with this and using novel studies emerging from several groups, including our own, we have illuminated its other vital function: desensitizing H_2_O_2_ signals. This occurs with mitochondrial proteins such as KGDH, PDH, complex I, and, most recently identified, DHODH. Notably, the desensitization of H_2_O_2_ signals may be a cell-wide function of glutathionylation as well given that recent evidence also demonstrated that it deactivates ROS production by xanthine oxidoreductase in rat liver cytoplasm in a GST-dependent manner [32]. Taken together, glutathionylation is a vital component of a cell’s signaling arsenal and serves as the foundation for redox communication.

## Data Availability

Data are available upon request from the corresponding author.
